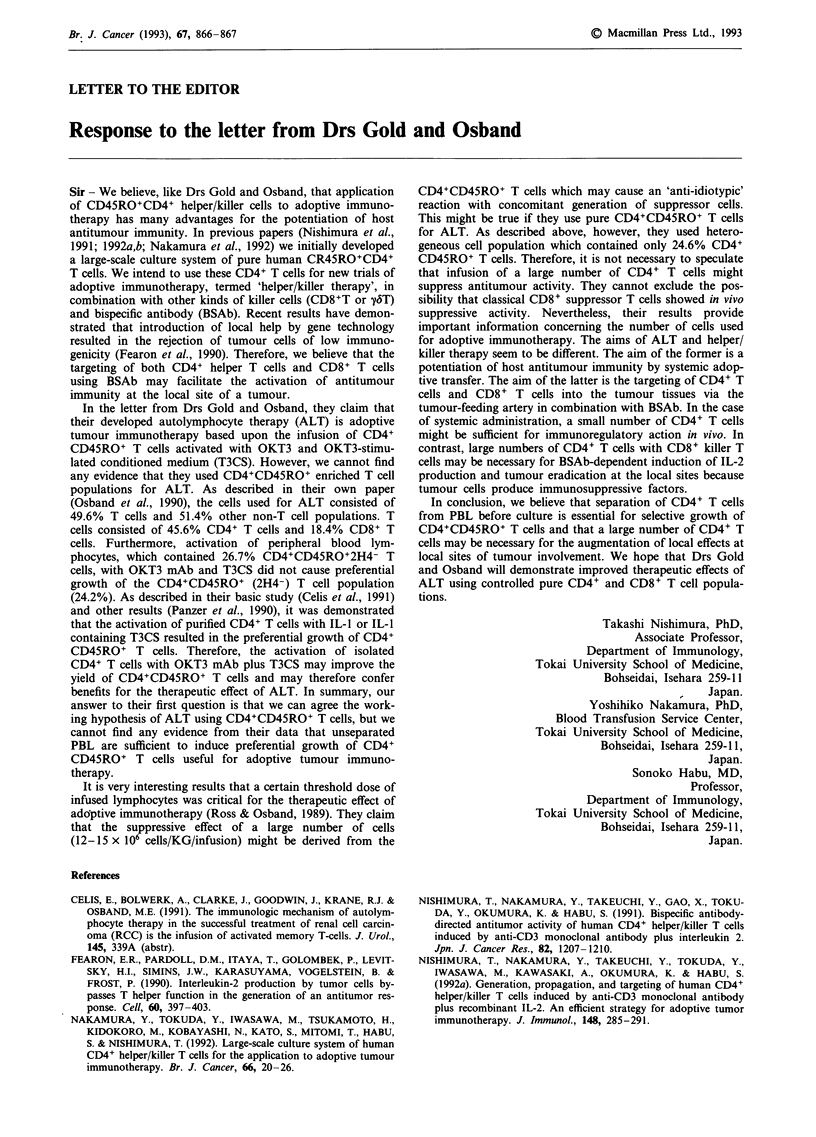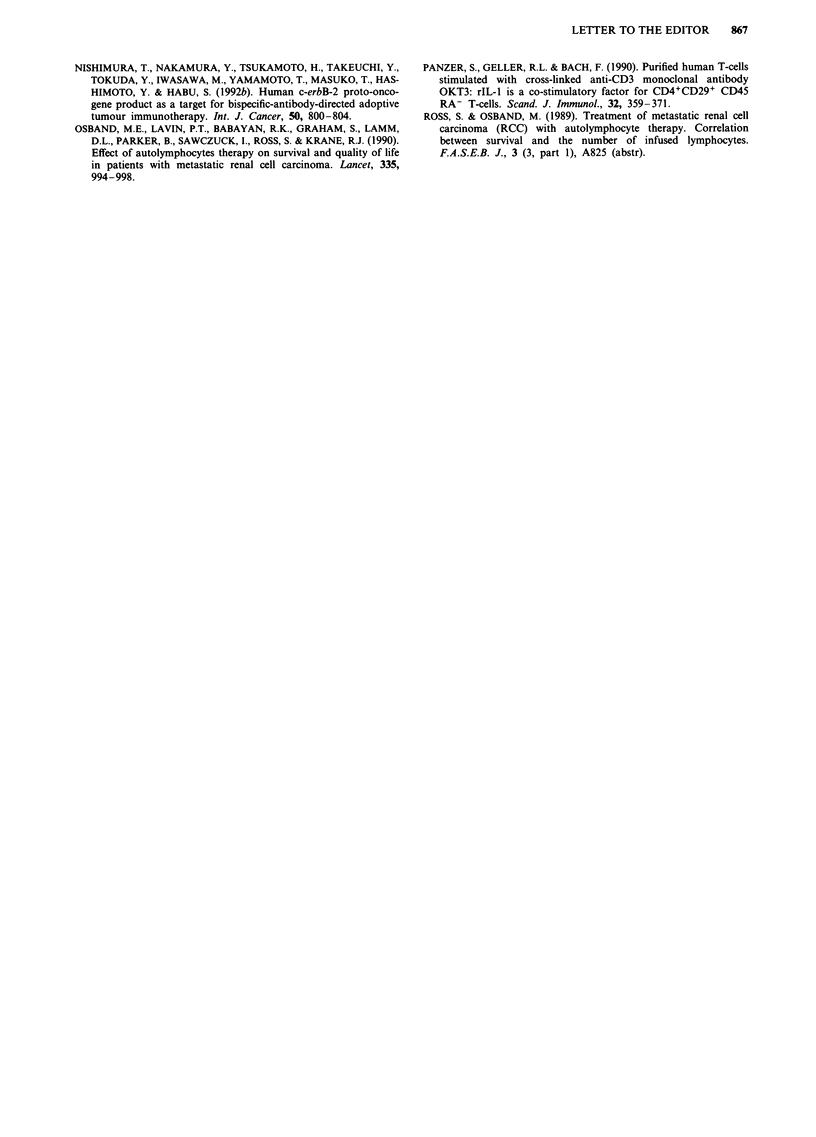# Response to the letter from Drs Gold and Osband

**Published:** 1993-04

**Authors:** Takashi Nishimura, Yoshihiko Nakamura, Sonoko Habu


Br. J. Cancer (1993), 67, 866-867 © Macmillan Press Ltd., 1993

### LETTER TO THE EDITOR

### Response to the letter from Drs Gold and Osband

**Sir** – We believe, like Drs Gold and Osband, that application of $CD45RO^+CD4^+$ helper/killer cells to adoptive immunotherapy has many advantages for the potentiation of host antitumour immunity. In previous papers (Nishimura *et al.*, 1991; 1992*a,b*; Nakamura *et al.*, 1992) we initially developed a large-scale culture system of pure human $CR45RO^+CD4^+$ T cells. We intend to use these $CD4^+$ T cells for new trials of adoptive immunotherapy, termed 'helper/killer therapy', in combination with other kinds of killer cells ($CD8^+T$ or $\gamma\delta T$) and bispecific antibody (BSAb). Recent results have demonstrated that introduction of local help by gene technology resulted in the rejection of tumour cells of low immunogenicity (Fearon *et al.*, 1990). Therefore, we believe that the targeting of both $CD4^+$ helper T cells and $CD8^+$ T cells using BSAb may facilitate the activation of antitumour immunity at the local site of a tumour.

In the letter from Drs Gold and Osband, they claim that their developed autolymphocyte therapy (ALT) is adoptive tumour immunotherapy based upon the infusion of $CD4^+$ $CD45RO^+$ T cells activated with OKT3 and OKT3-stimulated conditioned medium (T3CS). However, we cannot find any evidence that they used $CD4^+CD45RO^+$ enriched T cell populations for ALT. As described in their own paper (Osband *et al.*, 1990), the cells used for ALT consisted of 49.6% T cells and 51.4% other non-T cell populations. T cells consisted of 45.6% $CD4^+$ T cells and 18.4% $CD8^+$ T cells. Furthermore, activation of peripheral blood lymphocytes, which contained 26.7% $CD4^+CD45RO^+2H4^-$ T cells, with OKT3 mAb and T3CS did not cause preferential growth of the $CD4^+CD45RO^+$ ($2H4^-$) T cell population (24.2%). As described in their basic study (Celis *et al.*, 1991) and other results (Panzer *et al.*, 1990), it was demonstrated that the activation of purified $CD4^+$ T cells with IL-1 or IL-1 containing T3CS resulted in the preferential growth of $CD4^+$ $CD45RO^+$ T cells. Therefore, the activation of isolated $CD4^+$ T cells with OKT3 mAb plus T3CS may improve the yield of $CD4^+CD45RO^+$ T cells and may therefore confer benefits for the therapeutic effect of ALT. In summary, our answer to their first question is that we can agree the working hypothesis of ALT using $CD4^+CD45RO^+$ T cells, but we cannot find any evidence from their data that unseparated PBL are sufficient to induce preferential growth of $CD4^+$ $CD45RO^+$ T cells useful for adoptive tumour immunotherapy.

It is very interesting results that a certain threshold dose of infused lymphocytes was critical for the therapeutic effect of adoptive immunotherapy (Ross & Osband, 1989). They claim that the suppressive effect of a large number of cells ($12-15 \times 10^6$ cells/KG/infusion) might be derived from the $CD4^+CD45RO^+$ T cells which may cause an 'anti-idiotypic' reaction with concomitant generation of suppressor cells. This might be true if they use pure $CD4^+CD45RO^+$ T cells for ALT. As described above, however, they used heterogeneous cell population which contained only 24.6% $CD4^+$ $CD45RO^+$ T cells. Therefore, it is not necessary to speculate that infusion of a large number of $CD4^+$ T cells might suppress antitumour activity. They cannot exclude the possibility that classical $CD8^+$ suppressor T cells showed *in vivo* suppressive activity. Nevertheless, their results provide important information concerning the number of cells used for adoptive immunotherapy. The aims of ALT and helper/killer therapy seem to be different. The aim of the former is a potentiation of host antitumour immunity by systemic adoptive transfer. The aim of the latter is the targeting of $CD4^+$ T cells and $CD8^+$ T cells into the tumour tissues via the tumour-feeding artery in combination with BSAb. In the case of systemic administration, a small number of $CD4^+$ T cells might be sufficient for immunoregulatory action *in vivo*. In contrast, large numbers of $CD4^+$ T cells with $CD8^+$ killer T cells may be necessary for BSAb-dependent induction of IL-2 production and tumour eradication at the local sites because tumour cells produce immunosuppressive factors.

In conclusion, we believe that separation of $CD4^+$ T cells from PBL before culture is essential for selective growth of $CD4^+CD45RO^+$ T cells and that a large number of $CD4^+$ T cells may be necessary for the augmentation of local effects at local sites of tumour involvement. We hope that Drs Gold and Osband will demonstrate improved therapeutic effects of ALT using controlled pure $CD4^+$ and $CD8^+$ T cell populations.

Takashi Nishimura, PhD,
Associate Professor,
Department of Immunology,
Tokai University School of Medicine,
Bohseidai, Isehara 259-11
Japan.
Yoshihiko Nakamura, PhD,
Blood Transfusion Service Center,
Tokai University School of Medicine,
Bohseidai, Isehara 259-11,
Japan.
Sonoko Habu, MD,
Professor,
Department of Immunology,
Tokai University School of Medicine,
Bohseidai, Isehara 259-11,
Japan.

### References

CELIS, E., BOLWERK, A., CLARKE, J., GOODWIN, J., KRANE, R.J. & OSBAND, M.E. (1991). The immunologic mechanism of autolymphocyte therapy in the successful treatment of renal cell carcinoma (RCC) is the infusion of activated memory T-cells. *J. Urol.*, **145**, 339A (abstr).

FEARON, E.R., PARDOLL, D.M., ITAYA, T., GOLOMBEK, P., LEVITSKY, H.I., SIMINS, J.W., KARASUYAMA, VOGELSTEIN, B. & FROST, P. (1990). Interleukin-2 production by tumor cells bypasses T helper function in the generation of an antitumor response. *Cell*, **60**, 397-403.

NAKAMURA, Y., TOKUDA, Y., IWASAWA, M., TSUKAMOTO, H., KIDOKORO, M., KOBAYASHI, N., KATO, S., MITOMI, T., HABU, S. & NISHIMURA, T. (1992). Large-scale culture system of human $CD4^+$ helper/killer T cells for the application to adoptive tumour immunotherapy. *Br. J. Cancer*, **66**, 20-26.

NISHIMURA, T., NAKAMURA, Y., TAKEUCHI, Y., GAO, X., TOKUDA, Y., OKUMURA, K. & HABU, S. (1991). Bispecific antibody-directed antitumor activity of human $CD4^+$ helper/killer T cells induced by anti-CD3 monoclonal antibody plus interleukin 2. *Jpn. J. Cancer Res.*, **82**, 1207-1210.

NISHIMURA, T., NAKAMURA, Y., TAKEUCHI, Y., TOKUDA, Y., IWASAWA, M., KAWASAKI, A., OKUMURA, K. & HABU, S. (1992*a*). Generation, propagation, and targeting of human $CD4^+$ helper/killer T cells induced by anti-CD3 monoclonal antibody plus recombinant IL-2. An efficient strategy for adoptive tumor immunotherapy. *J. Immunol.*, **148**, 285-291.

NISHIMURA, T., NAKAMURA, Y., TSUKAMOTO, H., TAKEUCHI, Y., TOKUDA, Y., IWASAWA, M., YAMAMOTO, T., MASUKO, T., HASHIMOTO, Y. & HABU, S. (1992*b*). Human c-*erb*B-2 proto-oncogene product as a target for bispecific-antibody-directed adoptive tumour immunotherapy. *Int. J. Cancer*, **50**, 800–804.

OSBAND, M.E., LAVIN, P.T., BABAYAN, R.K., GRAHAM, S., LAMM, D.L., PARKER, B., SAWCZUCK, I., ROSS, S. & KRANE, R.J. (1990). Effect of autolymphocytes therapy on survival and quality of life in patients with metastatic renal cell carcinoma. *Lancet*, **335**, 994–998.

PANZER, S., GELLER, R.L. & BACH, F. (1990). Purified human T-cells stimulated with cross-linked anti-CD3 monoclonal antibody OKT3: rIL-1 is a co-stimulatory factor for $CD4^+CD29^+$ CD45 $RA^-$ T-cells. *Scand. J. Immunol.*, **32**, 359–371.

ROSS, S. & OSBAND, M. (1989). Treatment of metastatic renal cell carcinoma (RCC) with autolymphocyte therapy. Correlation between survival and the number of infused lymphocytes. *F.A.S.E.B. J.*, **3** (3, part 1), A825 (abstr).